# Transcranial Direct Current Stimulation Over Dorsolateral Prefrontal Cortex Modulates Risk-Attitude in Motor Decision-Making

**DOI:** 10.3389/fnhum.2019.00297

**Published:** 2019-09-06

**Authors:** Keiji Ota, Masahiro Shinya, Kazutoshi Kudo

**Affiliations:** ^1^Department of Psychology, New York University, New York, NY, United States; ^2^Institute of Engineering, Tokyo University of Agriculture and Technology, Tokyo, Japan; ^3^Japan Society for the Promotion of Science, Tokyo, Japan; ^4^Department of Human Sciences, Graduate School of Integrated Arts and Sciences, Hiroshima University, Hiroshima, Japan; ^5^Laboratory of Sports Sciences, Department of Life Sciences, Graduate School of Arts and Sciences, The University of Tokyo, Tokyo, Japan; ^6^Interfaculty Initiative in Information Studies, Graduate School of Interdisciplinary Information Studies, The University of Tokyo, Tokyo, Japan

**Keywords:** aim point, experience-based decision-making, inhibitory control, reward function, risk-taking behavior, non-invasive brain stimulation

## Abstract

Humans often face situations requiring a decision about where to throw an object or when to respond to a stimulus under risk. Several behavioral studies have shown that such motor decisions can be suboptimal, which results from a cognitive bias toward risk-seeking behavior. However, brain regions involved in risk-attitude of motor decision-making remain unclear. Here, we investigated the role of the dorsolateral prefrontal cortex (DLPFC) in risky motor decisions using transcranial direct current stimulation (tDCS). The experiment comprised a selective timing task requiring participants to make a continuous decision about the timing of their response under the risk of no rewards. The participants performed this task twice in a day: before and while receiving either anodal stimulation over the right DLPFC with cathodal stimulation over the left DLPFC (20 min, 2 mA), cathodal stimulation over the right DLPFC with anodal stimulation over the left DLPFC, or sham stimulation. In line with previous studies, their strategies before the stimulation were biased toward risk-seeking. During anodal stimulation over right DLPFC with cathodal stimulation over left DLPFC, participants showed a more conservative strategy to avoid the risk of no rewards. The additional experiment confirmed that tDCS did not affect the ability of timing control regarding the time intervals at which they aimed to respond. These results suggest a potential role for the DLPFC in modulating action selection in motor decision-making under risk.

## Introduction

Humans encounter many decision-making problems with different sources of uncertainty in daily life. A decision-making problem has been typically modeled as a mathematical formula, Σ*P*(*x*_*i*_)⋅ *x*_*i*_, where *x*_*i*_ represents the potential outcomes and *P*(*x*_*i*_) represents the corresponding probabilities of outcome occurrence; i.e., $\sum_{i=1}^{n}P\,\left(x_{i}\right)=1$ (Wu et al., 2009). For example, a problem of an economic decision-making task gives a decision-maker a choice between a lottery (0.5, $100; 0.5, $0) and a second lottery (1.0, $45). The first lottery indicates a 50–50 chance of winning $100 or nothing, whereas the second lottery indicates a guaranteed win of $45. This type of decision-making problem requires a discrete choice among a limited number of options (between two choices in this case, Knoch et al., 2006a; Fecteau et al., 2007a; Ye et al., 2015). A decision-maker also knows the information regarding the reward probabilities *P*(*x*_*i*_) because it is explicitly provided (Wu et al., 2009). A risk-averse decision-maker may prefer the second lottery (the sure win) to avoid risk even though the expected payoff is lower. Another type of decision-making problem is a motor decision task. It can be considered to have a parallel mathematical framework: ∫ *P*(*x*| *a*)⋅ *xdx*, where *x* represents a potential outcome and *P*(*x*| *a*) represents the probability that an action *a* leads an outcome *x*. Because in motor decision-making there are a theoretically infinite number of outcomes *x* caused by a specific action *a*, an integral equation rather than a summation is used. For example, a tennis player has a seemingly infinite number of options for aiming (aiming being an action) in a tennis court. For a given aim point, the actual location where the ball lands (the outcome of the action) could vary with every shot due to the inherent noise of the motor system. Unlike the economic decision, the probability *P*(*x*| *a*) is not explicitly given to the decision-maker. Therefore, the decision-maker must estimate how accurately he/she can hit the aim point based on their experience with the task. This type of decision-making problem requires a continuous choice of where, when, and how to act (in this case, where to aim in a tennis court) on the basis of the previous experience with a motor task (implicit probability information in the motor system) (Trommershäuser et al., 2008; Wu et al., 2009; Braun et al., 2011). If the player has a high accuracy, the ideal aim point should be close to the edge of the line. If the accuracy is low, he/she should aim more inner side of the line to avoid hitting the ball outside the line.

Motor decisions have been recently studied in a rich paradigm of simple experimental tasks. These studies have suggested that, under the symmetric reward structure in which constant gain came with a risk of penalty, human performance is consistent with the performance of a risk-neutral decision maker who maximizes the expected payoff (Trommershäuser et al., 2003a,b, 2005, 2008; Hudson et al., 2012). On the other hand, under the asymmetric reward structure in which higher gain came with a risk of penalty, humans tended to select a risky motor plan rather than the optimal motor plan (Wu et al., 2006; O’Brien and Ahmed, 2013; Ota et al., 2015, 2016). The violation of risk-neutrality has also been confirmed in a motor task involving a speed–accuracy trade-off (Nagengast et al., 2011b). We previously investigated whether the risk-neutral motor decision for maximizing the expected payoff can be reinforced by repetitive practice with reward feedback. We found that risk-neutral decisions appeared difficult to learn because participants’ risk-seeking or risk-averse behavior remains consistent even after 9 days of practice comprising 2,250 trials (Ota et al., 2016). Moreover, humans tend to be more risk-seeking when making motor decisions than economic decisions (Wu et al., 2009). Despite such behavioral evidence, little is known about the neural substrates underlying risk-attitude in motor decision-making.

Although research on the neural correlates of motor decision-making is in its early stages (Wu et al., 2011), it is assumed that the process of motor decision-making is supported by a distributed network of brain regions including the medial prefrontal cortex (mPFC), dorsolateral prefrontal cortex (DLPFC), anterior cingulate cortex (ACC), primary motor cortex (M1), lateral intraparietal cortex (LIP), and striatum (for a review, see Wu et al., 2015). The LIP is known to be involved in the accumulation of sensory information over time (Roitman and Shadlen, 2002). The mPFC represents implicit probability information produced by motor uncertainty (Wu et al., 2011) and reflects subjective value across different types of rewards (Levy and Glimcher, 2012). The DLPFC has been suggested as controlling behavior on the basis of reward information (Wallis and Miller, 2003), past outcomes, and previous decisions (Barraclough et al., 2004) which are encoded in this area. Activity in the DLPFC is specifically associated with decision-making in which the probability of an outcome is unknown (Krain et al., 2006). To gain further insight into the neural mechanism for motor decisions, it is crucial to identify brain regions causally involved in this function; however, such evidence remains missing.

In economic decision-making, it has been shown that DLPFC is causally involved in risk-taking behavior (Knoch et al., 2006a; Fecteau et al., 2007a; Ye et al., 2015; Huang et al., 2017; Luo et al., 2017). For instance, the deactivation of the right DLPFC induced by low-frequency repetitive transcranial magnetic stimulation (rTMS) increased the choice of high-risk over low-risk lottery (Knoch et al., 2006a). A transcranial direct current stimulation (tDCS) is another way to examine the causality of a brain region. In tDCS, cortical excitability in the stimulated area is increased by anodal stimulation (Nitsche and Paulus, 2000, 2001) and decreased by cathodal stimulation (Ardolino et al., 2005). These polarity-specific excitability shifts are thought to relate to the modulation of resting membrane potential (Purpura and McMurtry, 1965; Nitsche et al., 2003), which can transiently change behavioral performance. In fact, Fecteau et al. (2007a) showed that right anodal/left cathodal tDCS over the DLPFC inhibited the choice of the high-risk lottery. Luo et al. (2017) also showed that right anodal/left cathodal tDCS over the DLPFC induced more conservative and fairer judgment, whereas right cathodal/left anodal stimulation induced more risky and unfair judgment. Huang et al. (2017) demonstrated that the activation of the left DLPFC by anodal tDCS led to risk-averse decisions in the frame of monetary gain, while the inhibition of the right DLPFC by cathodal tDCS led to risk-seeking decisions in the frame of monetary loss.

In light of the evidence above, we directed our attention to the possible role of the DLPFC in motor decision-making. A problem of motor decision-making requires a continuous choice on the basis of implicit probability information (Trommershäuser et al., 2008; Wu et al., 2009; Braun et al., 2011). Given the different types of decision-making required for motor versus economic tasks, it is not clear whether the DLPFC is also involved with motor decision-making. In the present study, a tDCS intervention over the DLPFC was performed during a selective timing task in which participants were required to make a continuous decision about the timing of their response under risk while considering inherent motor noise. If the DLPFC is a common neural substrate underlying motor and economic decisions, a tDCS intervention over the DLPFC would alter the participant’s motor decisions. Given the evidence that bilateral tDCS over the DLPFC, but not unilateral tDCS of the right or left DLPFC, has a significant effect on modulating risk-taking behavior in economic decision tasks (Fecteau et al., 2007b), we performed bilateral stimulation over the DLPFC. In Experiment 1, we demonstrate that right anodal/left cathodal stimulation over the DLPFC inhibits risky motor decisions. In Experiment 2, we demonstrate that the same stimulation protocol does not affect response time under symmetric gain function where higher gain does not come with a risk of penalty, which suggests that tDCS is unlikely to influence the ability to accurately respond at the time that participants intend. Taken together, the obtained results suggest a potential causal role for the DLPFC in motor decision-making.

## Materials and Methods

### Participants

Thirty healthy right-handed adults were recruited using a recruitment flyer posted at The University of Tokyo. We confirmed that none of the participants had a history of neurological or psychiatric disorders based on their reports. All 30 participants were unaware of the purpose of the experiment and were naive to tDCS and to the experimental task. Experiment 1 was performed on 18 participants (11 males, 7 females; mean age 20.6 ± 2.3 years), and Experiment 2 was performed on 12 participants (10 males, 2 females; mean age 18.8 ± 0.6 years). The sample size was determined according to previous studies that confirmed a decrease in the risk-taking behavior by tDCS in samples of over 10 participants (Fecteau et al., 2007a,b).

### Experimental Task and Condition

**FIGURE 1:**
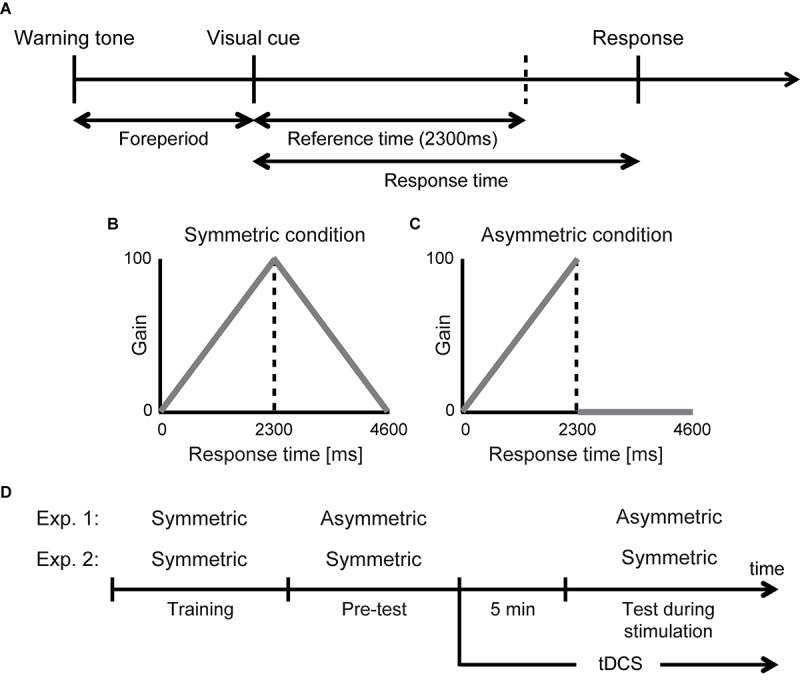
Experimental design and stimulation protocol. **(A)** Selective timing task. The participants were required to give a key press response after a foreperiod interval. The reference time was set at 2300 ms. In each trial, the response time was translated into a particular gain based on the following gain functions. **(B)** Symmetric condition. Higher gain was given as they could respond closer to the reference time. **(C)** Asymmetric condition. Within the reference time, they could receive higher gain as they responded closer to the reference time but they received no gain if they responded after the reference time. **(D)** Experimental design. Three experimental blocks (training, pre-test, test during stimulation) were conducted per stimulation protocol. There were three stimulation protocols (anodal stimulation over right DLPFC with cathodal stimulation over left DLPFC, cathodal stimulation over right DLPFC with anodal stimulation over left DLPFC, and sham stimulation). The participants performed the asymmetric condition or the symmetric condition receiving either one during stimulation. Each protocol was separated by 1 week. tDCS was started 5 min before the task in the test during stimulation block began and lasted for 15 min thereafter. In sham stimulation, tDCS was delivered only for an initial 40 s.

We used a selective timing task under risk based on our previous studies (Ota et al., 2015, 2016). In this task, we found that the risk-seeking strategy was selected. In the time sequence of our experimental task (Figure 1A), first, a warning tone was sounded to prepare the participants for an upcoming trial. A random foreperiod interval (800–1200 ms) preceded a visual cue presented on a computer screen (14 inches, refresh frequency 60 Hz) as a signal to inform the participants of a start. The reference time was set at 2300 ms after the onset of the visual cue. The participants pressed a button after the presentation of the visual cue. The response time (button press time – onset of a visual cue) was recorded in each trial.

The one-trial gain was determined by a gain function that translated the response time to a certain number of points. We prepared two different gain function conditions. First, in the symmetric condition with a symmetric gain function (Figure 1B), the participants received a gain that was a positive linear function of the response time when they responded earlier than the reference time. However, a response later than the reference time incurred a gain that was a negative linear function of the response time. Second, in the asymmetric condition with an asymmetric gain function (Figure 1C), the one-trial gain linearly rose as the reference time approached similar to the symmetric condition but plunged to zero thereafter. When they responded after the reference time and scored zero gain (0 points), an unpleasant alarm and a flashing red ramp appeared on the screen, signaling a mistrial.

The participants were informed by a visual and verbal explanation about the structure of the gain functions prior to running of each condition. In each trial, the relative response time (response time – reference time), the one-trial gain, and the cumulative total gain were given as performance feedback. Participants were instructed to maximize the total gain under each condition.

### Experimental Design

#### Experiment 1

We conducted three experimental blocks per day: *training* for providing the participants an opportunity to adapt the selective timing task, pre-test for testing the participant’s risk-attitude before the stimulation, and *test during stimulation* for testing risk-attitude changes induced by the stimulation. In Figure 1D, the experimental design for a single stimulation protocol is demonstrated. In Experiment 1, the training comprised the symmetric condition, the pre-test comprised the asymmetric condition, and the test during stimulation comprised the asymmetric condition. In the training, the pre-test, and the test during stimulation, there were 100, 50, and 50 trials, respectively. The participants came to the laboratory three times separated by 1 week and performed the selective timing task with either one of three stimulation protocols.

#### Experiment 2

We also performed Experiment 2 as a control experiment where the participants performed the symmetric condition in all of the experimental blocks. In this experiment, we aimed to investigate two potential confounds caused by stimulation. The first confound is that tDCS may influence the ability to accurately respond at the time that participants intend and simply reduce participants’ response times rather than affecting their risk-attitude. The second confound is that tDCS may affect temporal variance and reduce the variance in response time. The risk-attitude in a motor task is determined by both the mean response time and response variance (see section “Model Assumptions”). The decrease of response variance also reduces the participants’ risk-attitude without shifting the response time. In the symmetric condition, the participants were required to respond just at the reference time (that is, 2300 ms). Therefore, both effects of tDCS on the ability to accurately respond at the intended time and temporal variance could be investigated because their aim point was controlled at the reference time. The number of trials and stimulation protocols was the same as Experiment 1.

### Transcranial Direct Current Stimulation

Direct current induced through saline-soaked sponge electrodes (surface = 5 cm × 7 cm) was delivered by a battery-driven constant current stimulator (DC-STIMULATOR Plus, neuroConn GmbH). Electrode sizes were determined according to similar previous studies (Fecteau et al., 2007a,b; Ye et al., 2015). Three different stimulation protocols were applied to the participants during task performance. Each protocol was separated by a period of 1 week to reduce the carry-over effect of the stimulation. Not only was the order of stimulation protocols balanced across the participants, but it was also double blinded for the participants and the experimenter. To do so, an assistant told the experimenter a secret code of the stimulation (true or sham) before the experiment. The participants randomly received either anodal and cathodal stimulation over the right and left DLPFC, respectively (R anodal/L cathodal), cathodal and anodal stimulation over the right and left DLPFC, respectively (R cathodal/L anodal), or sham stimulation. We identified the location of the DLPFC using the international EEG 10–20 system. This method of DLPFC localization has been confirmed as a relatively accurate method in comparison with localization by a neuro-navigation technique (Herwig et al., 2003). For the R anodal/L cathodal condition, the anode electrode was placed over F4 and the cathode electrode was placed over F3. For the R cathodal/L anodal condition, the polarity was reversed. After the pre-test, we placed the electrodes on the participant’s scalp and secured them over the scalp with soft tape. tDCS started 5 min before the timing task began and was delivered during the entire 15-min course of the task (Figure 1D). A linear fade in/fade out of 10 s was applied. The participants gazed at a fixation point for the first 5 min. The intensity of stimulation was 2 mA. The stimulus duration and intensity were decided based on previous studies (Fecteau et al., 2007a,b). For sham stimulation, both electrodes were placed over either F3 or F4, but tDCS lasted only for an initial 40 s. The average (±standard deviation) impedance across the participants was 3.2 (±1.6) k ohms. No participants reported any side effects such as pain, itching, or headache after the experiment.

### Model Assumptions

**FIGURE 2:**
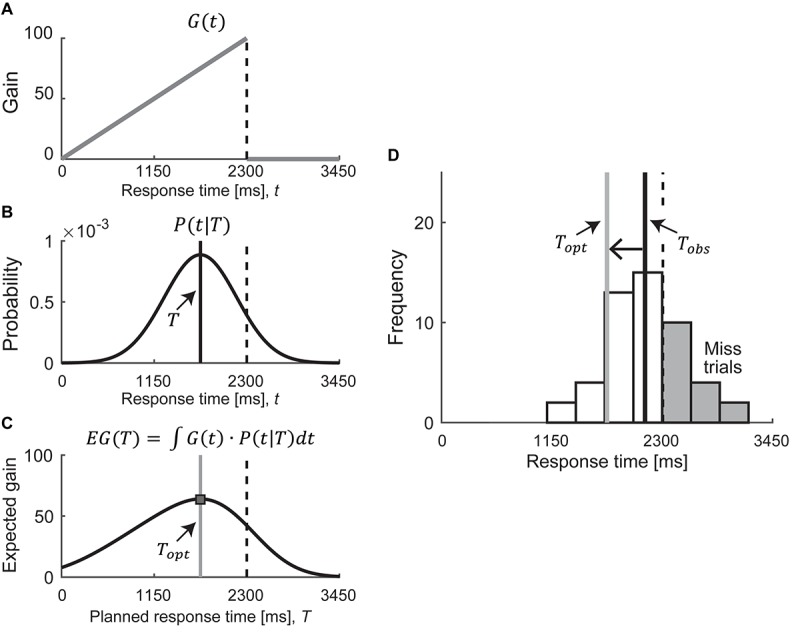
Model assumptions. **(A–C)** Calculation of the optimal mean response time in asymmetric condition. We modeled the optimal mean response time as $T_{o\,p\,t}\,\left(\sigma \right)=\underset{T}{\text{argmax}}E\,G\,\left(T\right)$. The expected gain can be calculated by integrating the gain function *G*(*t*) with the probability distribution of response time *P*(*t*|*T*), as a function of the planned response time *T*. The optimal mean response time (shown as a gray solid line) is defined as the response time that maximizes the expected gain. **(D)** Example of response histogram for risk-seeking strategy in asymmetric condition. In this example, the observed mean response time (shown as a black solid line) is slower than the optimal mean response time (shown as a gray solid line), which leads to too many mistrials (shown in the gray bars). Therefore, this strategy is classified as risk-seeking. A one-way left arrow shows the difference between *T*_*obs*_ and *T*_*opt*_. We defined this difference as the risk-attitude.

We estimated the optimal mean response time that maximizes the expected gain based on statistical/Bayesian decision theory (Berger, 1985; Trommershäuser et al., 2008; Maloney and Zhang, 2010) to quantify a participant’s risk-attitude. Bayesian decision theory uses the gain function and the probability density function. In this study, the gain functions correspond to the asymmetric condition (Figure 2A). The probability density function corresponds to the probability distribution of response time *P*(*t*| *T*), which we assumed as a Gaussian distribution with mean *T* and SD σ as follows:

(1) $$P\,\left(t|T\right)=\frac{1}{\sqrt{2\,\pi \,\sigma^{2}}}\,\exp\left[-\frac{{\left(t-T\right)}^{2}}{2\,\sigma^{2}}\right]$$

where *T* is the planned response time, *t* is the executed response time, and σ^2^ is the variability of response time (Figure 2B and Supplementary Figures 2, 3). Once we obtained a participant’s response variance σ^2^, we could calculate the expected gain *EG* as a function of the planned response time *T* by integrating the gain function *G*(*t*) over the probability distribution *P*(*t*| *T*) as follows:

(2) $$E\,G\,\left(T\right)=\int_{-\infty }^{\infty }G\,\left(t\right)\cdot P\,\left(t|T\right)\,𝑑t$$

This expected gain function, which is obtained by integrating the gain function in the asymmetric condition over the probability distribution, is illustrated in Figure 2C. We defined the optimal mean response time *T*_*opt*_—illustrated as a gray solid line in Figure 2C —as the response time that maximized the expected gain:

(3) $$T_{o\,p\,t}\,\left(\sigma \right)=\underset{T}{\text{argmax}}E\,G\,\left(T\right)$$

We then compared this optimal mean response time *T*_*opt*_ with the observed mean response time *T*_*obs*_. If *T*_*obs*_ corresponded to *T*_*opt*_, it indicated that the participants followed a risk-neutral strategy for their given response variance. In contrast, in the asymmetric condition, if *T*_*obs*_ were slower than *T*_*opt*_ (closer to the reference time than *T*_*opt*_), the participants adopted a suboptimal risk-seeking strategy (Figure 2D). The risk-seeking strategy indicated that the participants sought a high one-trial reward with a high probability of failure. As shown in gray bars in Figure 2D, it produces many mistrials. If *T*_*obs*_ were faster than *T*_*opt*_ (further from the reference time than *T*_*opt*_), the participants adopted a suboptimal risk-averse strategy. The risk-averse strategy indicated that the participants sought a low one-trial reward and avoided a high probability of failure. Both strategies were suboptimal in terms of maximizing the expected gain. We defined the participant’s risk-attitude as the difference between the observed mean response time and the optimal mean response time, *T*_*obs*_ – *T*_*opt*_. For a detailed description of our procedure and model assumptions, see Ota et al. (2015, 2016).

### Data Analysis

In each trial, we recorded the response time from the onset of the visual cue to the key press time and defined it as the response time. Trials in which the response times exceeded ± 2.5 SD from the mean were excluded from the analysis as outliers. As a result, we removed 172 of a total of 10,800 trials (1.6%) in Experiment 1 and 111 of a total of 7,200 trials (1.5%) in Experiment 2. *Post hoc* tests with Bonferroni correction were performed in the case of significant results following analysis of variance (ANOVA). *P* < 0.05 was considered statistically significant. Cohen’s *d* and η^2^ were calculated as an index of effect size. SPSS software (IBM, Armonk, NY, United States, version 24.0) was used for all the statistical tests.

## Results

**FIGURE 3:**
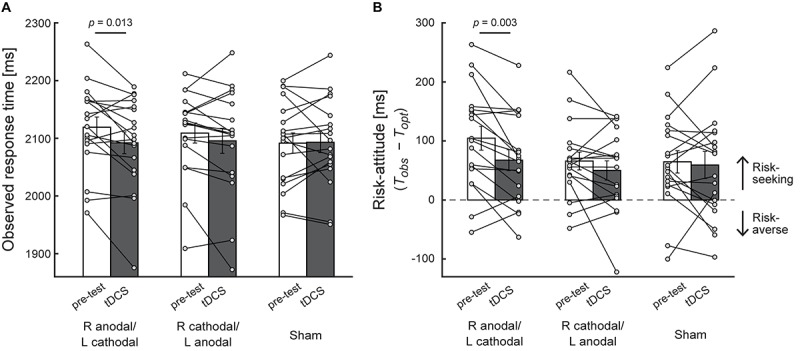
Behavioral performance in the asymmetric condition. **(A)** The average observed mean response time is plotted. The *X*-axis indicates experimental blocks (pre-test and test during stimulation) in each stimulation protocol. R anodal/L cathodal = anodal transcranial direct current stimulation (tDCS) over the right DLPFC and cathodal tDCS over the left DLPFC. R cathodal/L anodal = cathodal tDCS over the right DLPFC and anodal tDCS over the left DLPFC. Compared with the pre-test, a significant decrease in the observed mean response time was found in R anodal/L cathodal stimulation. **(B)** The average risk-attitude (*T*_*obs*_ − *T*_*opt*_) across the participants. A significant decrease was found in R anodal/L cathodal stimulation, which suggests that the risk-seeking strategy in motor decisions was modulated by bilateral tDCS over the DLPFC. For calculation of risk-attitude, see Figure 2. Each circle represents the individual data. Error bar indicates the standard error of the mean in both panels.

The results in the test during stimulation of Experiment 1 (asymmetric condition) were obtained from the double-blind assessment of the stimulation type. In Figures 3A,B, we illustrate the average observed mean response time and the average risk-attitude across the participants with each of three stimulation protocols, respectively. We first observed that before receiving current stimulation (pre-test), the participants adopted a suboptimal risk-seeking strategy as shown in previous studies (O’Brien and Ahmed, 2013; Ota et al., 2015, 2016). The risk-attitude value [R anodal/L cathodal: 105 ± 20 ms (mean ± standard error of mean), R cathodal/L anodal: 66 ± 15 ms, Sham: 65 ± 19 ms] was significantly larger than 0 in the pre-test of all three stimulus protocols (two-tailed paired *t*-test: *ts* [17] > 3.43, *ps* < 0.01, white bars in Figure 3B). In the pre-test, the observed mean response time was 2119 ± 18 (mean ± sem) ms for R anodal/L cathodal, 2109 ± 17 ms for R cathodal/L anodal, and 2092 ± 18 ms for sham (white bars in Figure 3A). To confirm that the initial pre-test performance did not differ across stimulus protocols, the one-way within-subject ANOVA was performed. The results showed no main effect of stimulus protocol for the observed mean response time [*F*(2,34) = 2.78, *p* = 0.076] and for risk-attitude [*F*(2,34) = 3.18, *p* = 0.054]. There were no significant differences among the pre-test of three stimulus protocols in the observed mean response time (*p*s > 0.068) and the risk-attitude (*p*s > 0.12).

To determine the effect of tDCS on motor decision-making, we performed two-way within-subject ANOVA using the observed mean response time as dependent variable. The result showed a significant interaction effect of experimental block and stimulation protocol [*F*(2,34) = 3.35, *p* = 0.047, η^2^ = 0.03]. The *post hoc* test revealed that, in R anodal/L cathodal condition, the observed mean response time in the test during stimulation (2,092 ± 19 ms, mean ± sem) was significantly shorter than that in the pre-test (2,119 ± 18 ms) (*p* = 0.013, *d* = 0.35, Figure 3A). Conversely, no significant change was found in the other two conditions (*p*s > 0.16, *d*s < 0.16). This decrease in the observed mean response time by R anodal/L cathodal stimulation also induced the decrease in risk-attitude value. Although we did not find a significant interaction effect of experimental block and stimulation protocol [*F*(2,34) = 2.24, *p* = 0.12, η^2^ = 0.02], a significant decrease of risk-attitude was found between the pre-test (105 ± 20 ms) and the test during stimulation (68 ± 18 ms) in R anodal/L cathodal condition (*p* = 0.003, *d* = 0.46, Figure 3B). There was no significant change in the other two conditions (*p*s > 0.21, *d*s < 0.24). These results suggest that, though the risk-attitude was still positive (that is, risk-seeking) during R anodal/L cathodal stimulation [two-tailed one sample *t*-test from 0: *t*(17) = 3.80, *p* = 0.001], the participant’s risk-attitude decreased and approached to risk-neutral by R anodal/L cathodal stimulation compared with the pre-test.

**FIGURE 4:**
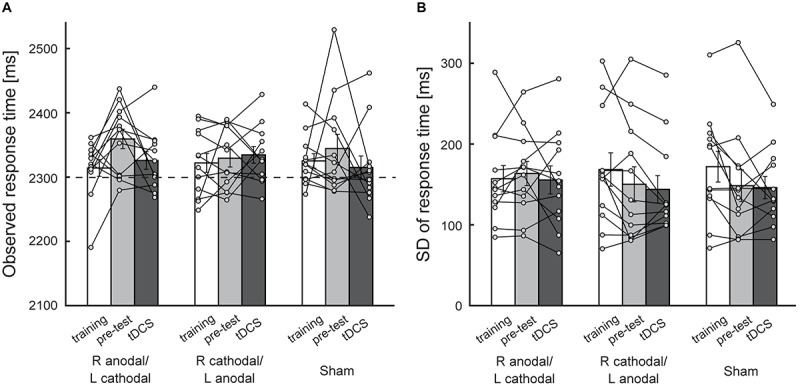
Behavioral performance in the symmetric condition. **(A)** The average observed mean response time across the participants is plotted. The *X*-axis indicates experimental blocks (training, pre-test, and test during stimulation) in each stimulation protocol. There were no significant differences between the pre-test and the test during stimulation in the R anodal/L cathodal stimulation condition, which suggests that tDCS did not affect response time when the aim point in response time was controlled at 2300 ms. See Supplementary Figure 4 for risk-attitude analysis in Experiment 2. **(B)** The average standard deviation of response time is plotted as an index of response variance. tDCS did not affect response variance. Each circle represents the individual data. Error bar indicates the standard error of the mean in both panels.

To investigate (1) whether tDCS simply reduced participants’ response time rather than affecting their risk-attitude and (2) whether tDCS attenuated their risk-attitude by reducing the variance in response time, we conducted Experiment 2 in which the participants performed the symmetric condition during the stimulation (Figures 1B,D). The average observed mean response time across the participants with each of three experimental blocks and each of three stimulation protocols is illustrated in Figure 4A. To obtain comparable results with Experiment 1, we performed two-way within-subject ANOVA using two experimental blocks (pre-test and test during stimulation) and three stimulation protocols as independent variables. Neither main effect of stimulation protocol [*F*(2,22) = 0.44, *p* = 0.65, η^2^ = 0.02] nor significant interaction [*F*(2,22) = 1.32, *p* = 0.29, η^2^ = 0.04] was found. There was no significant difference between the test during stimulation (2,326 ± 14 ms, mean ± sem) and the pre-test (2,359 ± 15 ms) in the R anodal/L cathodal condition (*p* = 0.078, Figure 4A). Because the participants also performed the symmetric condition during the training, we compared the test during stimulation (2,326 ± 14 ms) with the training (2,314 ± 13 ms) and found no significant difference [two-tailed paired *t*-test: *t*(11) = –0.64, *p* = 0.54, Figure 4A]. In Figure 4B, the average standard deviation of the response time across the participants is illustrated. Similarly, two-way (2 blocks × 3 stimulations) within-subject ANOVA revealed no main effect of stimulation protocol [*F*(2,22) = 1.11, *p* = 0.35, η^2^ = 0.05] and no significant interaction [*F*(2, 22) = 0.06, *p* = 0.94, η^2^ = 0.00]. In the R anodal/L cathodal condition, there was no significant difference between the test during stimulation (156 ± 17 ms) and the pre-test (164 ± 15 ms) (*p* = 0.51, Figure 4B) nor between the test during stimulation and the training (157 ± 16 ms) [two-tailed paired *t*-test: *t*(11) = 0.09, *p* = 0.93, Figure 4B]. These results validate the assumption that our stimulation protocols did not modulate the response time and the response variance when the aim point was controlled at the reference time. Therefore, the obtained results in Experiments 1 were likely to be induced by the modulation of the decision process that attempts to aim further from the penalty, rather than by the modulation of the temporal process.

## Discussion

Emerging evidence has identified sub-optimal and risk-sensitive behavior in motor control tasks requiring a decision about where to aim under an asymmetric gain function (Wu et al., 2006; O’Brien and Ahmed, 2013; Ota et al., 2015, 2016) or a decision involving a speed–accuracy trade-off (Nagengast et al., 2011b). In the present study, we investigated the role of the DLPFC in risk-sensitive motor decision-making. In Experiment 1, we conducted a within-participant experiment in which participants were required to perform the asymmetric condition for two blocks (pre-test and test during stimulation). Consistent with previous studies, we found that the risk-seeking strategy was adopted in the pre-test in which the observed mean response time was larger than the optimal mean response time. However, the observed mean response time became shorter during the R anodal/L cathodal stimulation condition. Therefore, the results suggest that the activation of the right DLPFC and the deactivation of the left DLPFC decreased risk-seeking behavior.

The causal role of the DLPFC in risk-taking behavior has been shown in economic decision tasks (Knoch et al., 2006a; Fecteau et al., 2007a; Ye et al., 2015; Huang et al., 2017; Luo et al., 2017). These studies asked participants to decide between two lotteries in which information on reward and reward probability was explicitly provided to participants. By contrast, in motor decision tasks, participants were required to make a continuous choice about where to aim and the reward probability depends on their experience of motor tasks (Trommershäuser et al., 2008; Wu et al., 2009; Braun et al., 2011). The participants therefore need to take implicit probability information (that is, their own response variance) into account for making a decision (Wu et al., 2009; Ota et al., 2016). Because the motor and economic tasks require different decision-making, it was possible that the DLPFC did not contribute to motor decision-making. What we revealed is that the intervention of the DLPFC altered the participant’s choice even when continuous decisions were made by relying on their implicit experience. This suggests that the DLPFC is a common neural substrate underlying motor decisions and economic decisions.

How can we explain the modulated risk-attitude by R anodal/L cathodal stimulation? One possibility is that tDCS modulated an inhibitory function toward upcoming rewards in the DLPFC. Fecteau et al. (2007a) found that R anodal/L cathodal stimulation over the DLPFC increased the choice of low-risk lottery involving small rewards over high-risk lottery involving large rewards. The same stimulation also decreases craving levels in substance-dependent patients (Boggio et al., 2008, 2010; Fecteau et al., 2014). In contrast to these inhibitory effects by the activation of the right DLPFC, the deactivation of the right DLPFC with low-frequency rTMS resulted in more frequently choosing the risky lottery (Knoch et al., 2006a) and an increase in the acceptance of unfair offers (van’t Wout et al., 2005; Knoch et al., 2006b). In our task, the risk-neutral response for maximizing the expected payoff is to accumulate an optimal one-trial gain for a given response variance. However, risk-seeking individuals are driven to aim close to the maximum gain rather than the optimal gain and this increases the percentage of mistrials (zero gain). Therefore, these risky behaviors might be inhibited by the activation of the inhibitory function in the DLPFC induced by R anodal/L cathodal stimulation.

Two confounding factors should be discussed to qualify our results. The first is whether tDCS affects temporal variance. The risk-attitude value in a motor task should decrease if the temporal variance decreases (Ota et al., 2015, 2016). In Experiment 2, we confirmed that bilateral stimulation had no effect on the SD of response time in the symmetric condition. This also suggests that the stimulation was not likely to affect the participant’s estimation of his/her own response variance. The second factor is whether tDCS affects the ability to accurately respond at the time that participants intend. It has been suggested that the DLPFC is involved in cognitive timing control (Koch et al., 2009). Previous studies have shown that low-frequency rTMS over the right DLPFC induced the misestimation of time periods in a seconds-range time reproduction task requiring the estimation of the presented time interval and the reproduction of the estimated intervals (Koch et al., 2003, 2007). In the time reproduction task, the presented time intervals varied from trial to trial. Jones et al. (2004) suggested that this misestimation of the time interval reflects an interference with the memory process that consolidates the presented time intervals in memory, rather than interference with the time clock process.

In the present task, however, the participants did not need to memorize the time interval because it was fixed (that is, 2300 ms). Indeed, under the symmetric condition in Experiment 2, R anodal/L cathodal stimulation did not induce the response time change compared with the pre-test and the training. Our stimulation protocols therefore were not likely to have disrupted the ability to accurately respond at the time that they intended. To address this issue further, we conducted a preliminary experiment for the additional asymmetric condition in which the participants had to respond later than the reference time (Supplementary Figure 1A). In this reversed asymmetric gain function, if tDCS over the DLPFC induced the risk-averse response style as in Experiment 1, the response time would be prolonged. Preliminary data showed that the observed mean response time during the R anodal/L cathodal stimulation (2,559 ± 38 ms, mean ± sem) was significantly slower than that in the pre-test (2,496 ± 34 ms) [two-tailed paired *t*-test: *t*(4) = −4.59, *p* = 0.010, *d* = −0.88, Supplementary Figure 1B]. Therefore, the change in response time observed in this study was better explained by the modulation of risk-attitude rather than the disruption in the response time.

This study has several limitations. First, some measurements of personality and emotional state should have been taken before and after the stimulation, since personality and emotional state could influence the evaluation of the outcomes and thus affect decision-making (Lerner et al., 2015). These measurements might help to understand what proportion of our results could be explained by stimulation-induced changes in personality and emotion. Second, we cannot distinguish whether the behavioral changes were caused by the combined stimulation of the DLPFC with the anodal right and cathodal left electrode or solely by the activation of the right DLPFC with the anodal electrode. A previous study suggested that because unilateral anodal stimulation over the right DLPFC was not sufficient to modulate risk-taking behavior, this effect is mediated by the relative balance of activity across the right and the left DLPFC (Fecteau et al., 2007b). At this point, we can speculate that the contralateral effects of stimulating the DLPFC in the opposite direction altered the motor decisions in this study.

Third, we cannot rule out the possibility that the neighboring regions such as the ventromedial prefrontal cortex (vmPFC)/ oribitofrontal cortex (OFC) interconnected with the DLPFC (Cavada et al., 2000) were influenced by tDCS and might have contributed to behavioral changes. The vmPFC/OFC is the overlapping region that represents subjective value across different types of rewards (Peters and Büchel, 2010; Levy and Glimcher, 2012). Indeed, tDCS-induced modulation in neural activity has been suggested to spread beyond the directly stimulated area to the neighboring cortex (Weber et al., 2014; Nakamura-Palacios et al., 2016) and even to the subcortical region (Tanaka et al., 2013; Weber et al., 2014; Nakamura-Palacios et al., 2016). Weber et al. (2014) stimulated the bilateral DLPFC with smaller electrodes (5 cm × 5 cm) used in this study. They showed that the stimulation decreased resting blood perfusion in orbitofrontal cortex and right caudate and also increased task-related activity in the ACC. Therefore, even with a smaller electrode size, the interconnectedness of the DLPFC with neighboring cortical and subcortical regions will create potential confounds—this is a methodological limitation that affects the vast majority of electrophysiological studies in the human brain. However, it should be mentioned that the DLPFC is not isolated, but is part of a network that governs decision-making together with the interconnected regions (Levy and Glimcher, 2012). In this sense, although stimulation-induced downstream mechanisms are unclear, our conclusion that tDCS intervention over the DLPFC modulates motor decisions remains valid. Future studies could better clarify this issue by using a centering montage that enables experimenters a more focal stimulation within the target area (Heise et al., 2016).

On a similar note, a lack of application of neuroimaging techniques is also a limitation in this study. tDCS has been developed as an effective tool for brain research. There are other techniques being developed that combine tDCS and fMRI or EEG, allowing experimenters to investigate stimulation-induced changes in BOLD signals (Antal et al., 2011, 2012), resting-state connectivity (Keeser et al., 2011), synchronization of brain waves (Polanía et al., 2011), and EEG power spectral density (Mangia et al., 2014). This study was a pilot study that applied tDCS to motor decision-making. We thus focused primarily on behavioral changes. However, issues with respect to the ambiguities of how brain activity patterns change during the motor decision task and how it led to a change in risk-attitude could be solved by the application of fMRI-tDCS or EEG-tDCS techniques.

## Conclusion

Despite the behavioral evidence of risk-attitude in motor decision tasks (Wu et al., 2006, 2009; Nagengast et al., 2011a,b; O’Brien and Ahmed, 2013; Ota et al., 2015, 2016), the neural mechanism of this function is insufficiently known. Although a study reported the neural correlates involving motor decisions (Wu et al., 2011), the brain regions causally involved in risk-attitude of motor decisions have not been identified as of yet. Our work sheds light on this issue. We found that participants’ risk-seeking behavior in a motor decision task decreased with the simultaneous activation of the right DLPFC and deactivation of the left DLPFC, demonstrating a potential causal role of the DLPFC in motor decision-making. A next step would be to investigate whether motor decisions are solely governed by the DLPFC or also by other brain areas. We propose future exploratory studies to target various brain regions for advancing the understanding of neural mechanisms underlying risk-attitude in motor decisions.

## Ethics Statement

This study was approved by the Ethics Committee of the Graduate School of Arts and Sciences, The University of Tokyo, and the methods were conducted in accordance with the approved guidelines. Signed informed consent was obtained from all participants.

## Author Contributions

KO, MS, and KK conceived and designed the experiments, analyzed the data, and interpreted the results. KO performed the experiments and drafted the manuscript. MS and KK revised the manuscript.

## Conflict of Interest Statement

The authors declare that the research was conducted in the absence of any commercial or financial relationships that could be construed as a potential conflict of interest.
