# Supplementary material for: Transcranial Direct Current Stimulation Over Dorsolateral Prefrontal Cortex Modulates Risk-Attitude in Motor Decision-Making
Source: Front Hum Neurosci. 2019 Sep 6;13:297. doi: 10.3389/fnhum.2019.00297 (PMC6743341; doi:10.3389/fnhum.2019.00297)
Supplement: Supplementary file 1 [file Table_1.docx]

Supplementary Material

**Transcranial direct current stimulation over dorsolateral prefrontal cortex modulates risk-attitude in motor decision-making**

Keiji Ota*, Masahiro Shinya, Kazutoshi Kudo*

*** Correspondence:** Keiji Ota, Email: [keiji.ota@nyu.edu](mailto:keiji.ota@nyu.edu)

Kazutoshi Kudo, Email: [kudo@idaten.c.u-tokyo.ac.jp](mailto:kudo@idaten.c.u-tokyo.ac.jp)

**This PDF file includes:**

Supplementary Figures. 1 to 4

# Additional experiment

We further conducted a preliminary experiment to determine whether tDCS affected the selection of response timing or the ability to accurately respond the intended time. In this experiment, we made another asymmetric gain function by flipping the asymmetric gain function used in Experiment 1 (Supplementary Figure 1A) and the participants was required to respond after the reference time. We hypothesized that if tDCS induced a risk-averse response style as in Experiment 1, the response time would be prolonged. Five participants (3 males, 2 females; mean age 22.2 ± 3.7 years) who were recruited from Experiment 1 came to the laboratory 1 week after the last stimulation in Experiment 1 had ended. The participants first performed the symmetric condition for 100 trials (training). They then performed this reversed asymmetric gain function condition for 50 trials without stimulation (pre-test) and again (50 trials) receiving R anodal/L cathodal stimulation over DLPFC. The anode electrode was placed over F4 and the cathode electrode was placed over F3. The stimulation duration and intensity were the same those in Experiment 1 & 2 (see, Methods). We found that the observed mean response time $T_{obs}$ in the test during stimulation (2559 ± 38 ms, mean ± sem) was significantly slower than that in the pre-test (2496 ± 34 ms) (two-tailed paired *t* test: *t* [4] = −4.59, *p* = 0.010, *d* = −0.79, Supplementary Figure 1B). We further found a change in the risk-attitude. The risk-attitude (herein, defined as $T_{opt}- T_{obs}$) showed positive values (Supplementary Figure 1C), indicating that the participants responded faster (closer to the reference time) than the optimal mean response time $T_{opt}$ (risk-seeking strategy). However, in the test during stimulation (4 ± 27 ms), the risk-attitude value was significantly lower than that in the pre-test (67 ± 27 ms) (two-tailed paired *t* test: *t* [4] = 3.48, *p* = 0.025, *d* = 1.06, Suppl. Fig. 1c). Although our sample was limited, we speculate that our stimulation affected the selection of response timing.


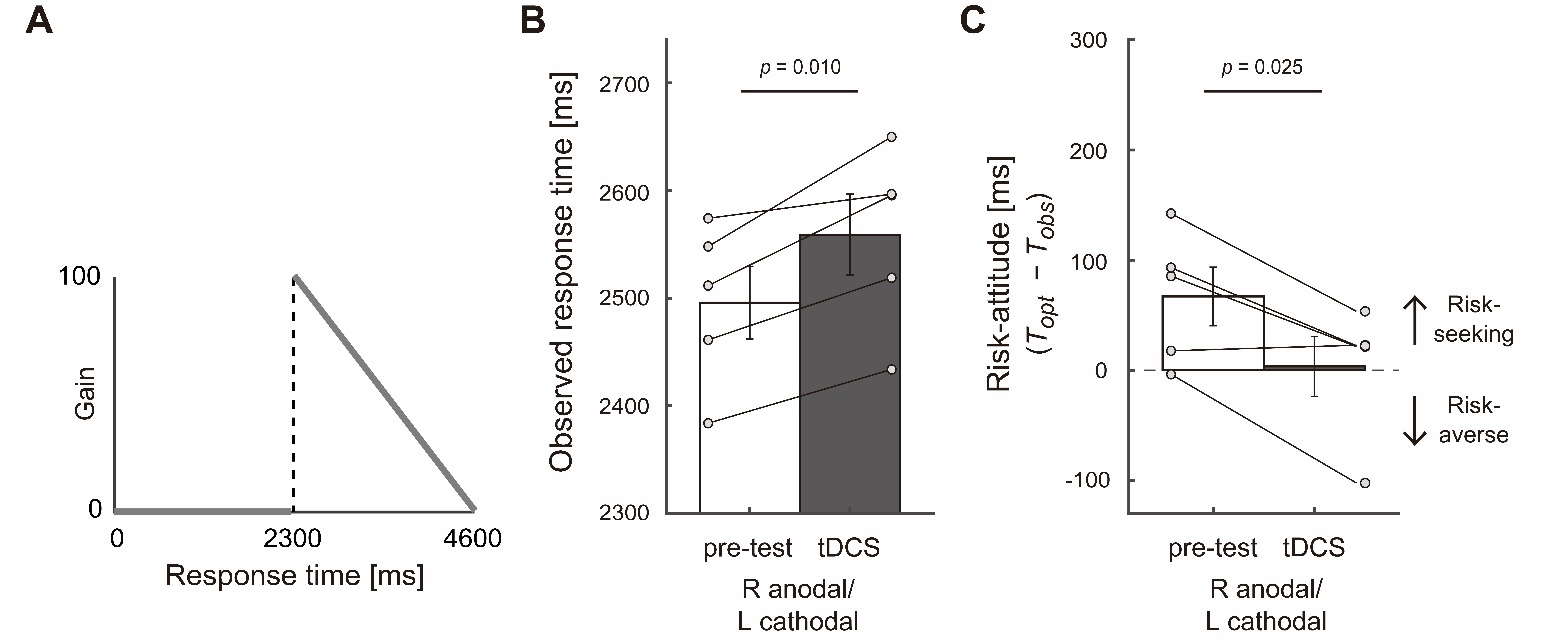


**Supplementary Figure 1.** (A) Another asymmetric condition. No gain was attributed if the participants responded before the reference time. After the reference time, the gain decreased as a linear function of response time. (B) The average observed mean response time across the participants is plotted in the pre-test and R anodal/L cathodal stimulation. (C) The average risk-attitude ($T_{opt}- T_{obs}$) across the participants. Each circle represents the individual data. Error bar indicates the standard error of the mean in both panels.

# Model Assumptions

In the model assumptions, we assumed that participant’s response time follows a Gaussian distribution. To confirm this, we plotted a quantile-quantile plot for response time data and conducted Kolmogorov-Smirnov test. Supplementary Figure 2A and B illustrate a Q-Q plot for the pre-test and the test during stimulation in right anodal/left cathodal condition in Experiment 1(asymmetric condition), respectively. Each panel indicates an individual subject and p-value shows a result of Kolmogorov-Smirnov test. The null hypothesis that response time follows a Gaussian distribution was not significantly rejected for 77.8 % (14/18) of participants in the pre-test and for 100% (18/18) in the test during stimulation. Supplementary Figure 3a&b illustrate a Q-Q plot for the pre-test and the test during stimulation in right anodal/left cathodal condition in Experiment 2 (symmetric condition), respectively. Similarly, the null hypothesis was not significantly rejected for 91.7 % (11/12) of participants in the pre-test and for 91.7% (11/12) in the test during stimulation. These results suggest that participant’s response time distributes according to a Gaussian distribution.


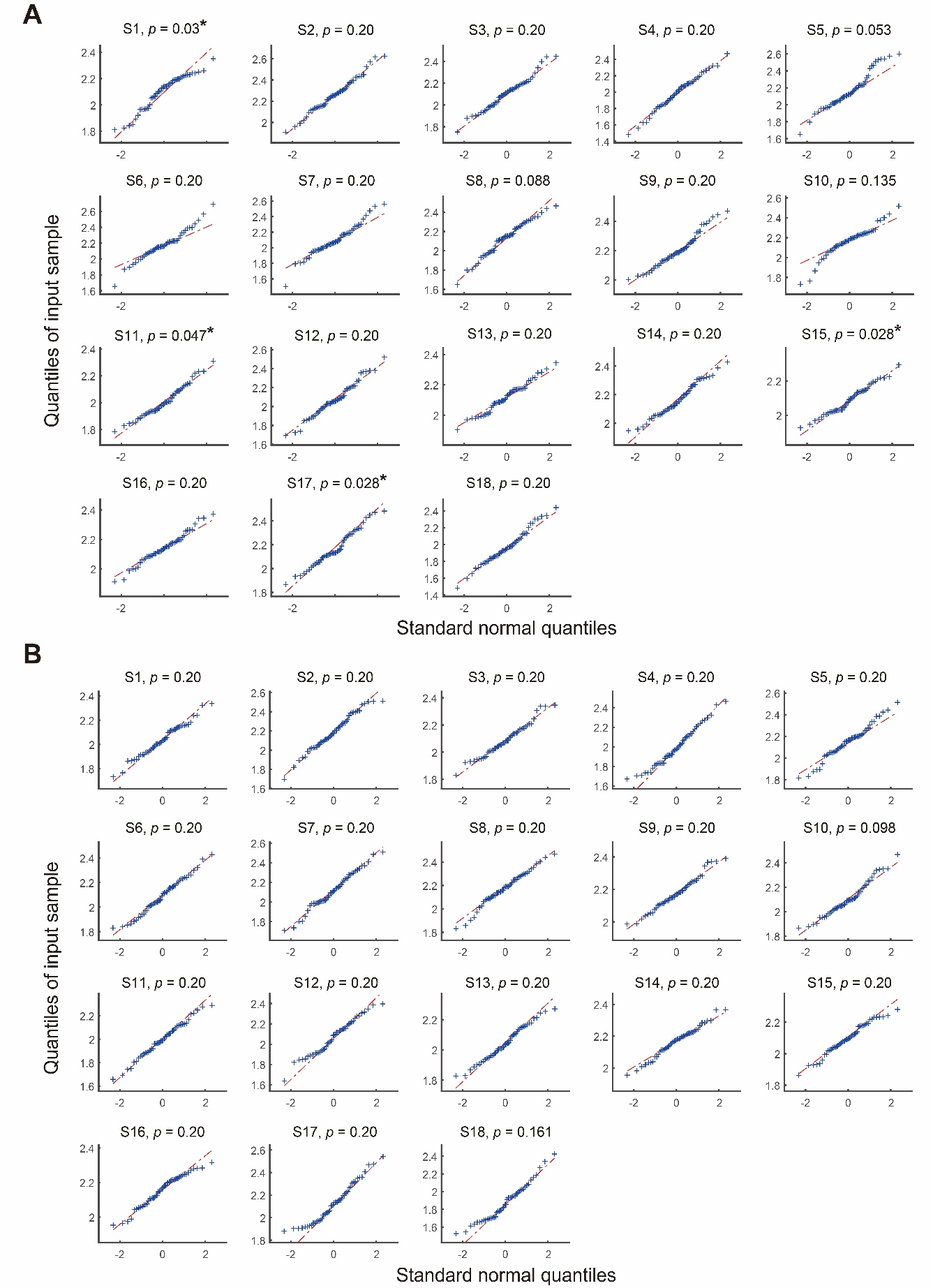
**Supplementary Figure 2.** Q-Q plot for the response time data and the result of Kolmogorov-Smirnov test in Experiment 1 (asymmetric condition). Pre-test (A) and test during stimulation (B) in right anodal/left cathodal condition.

**
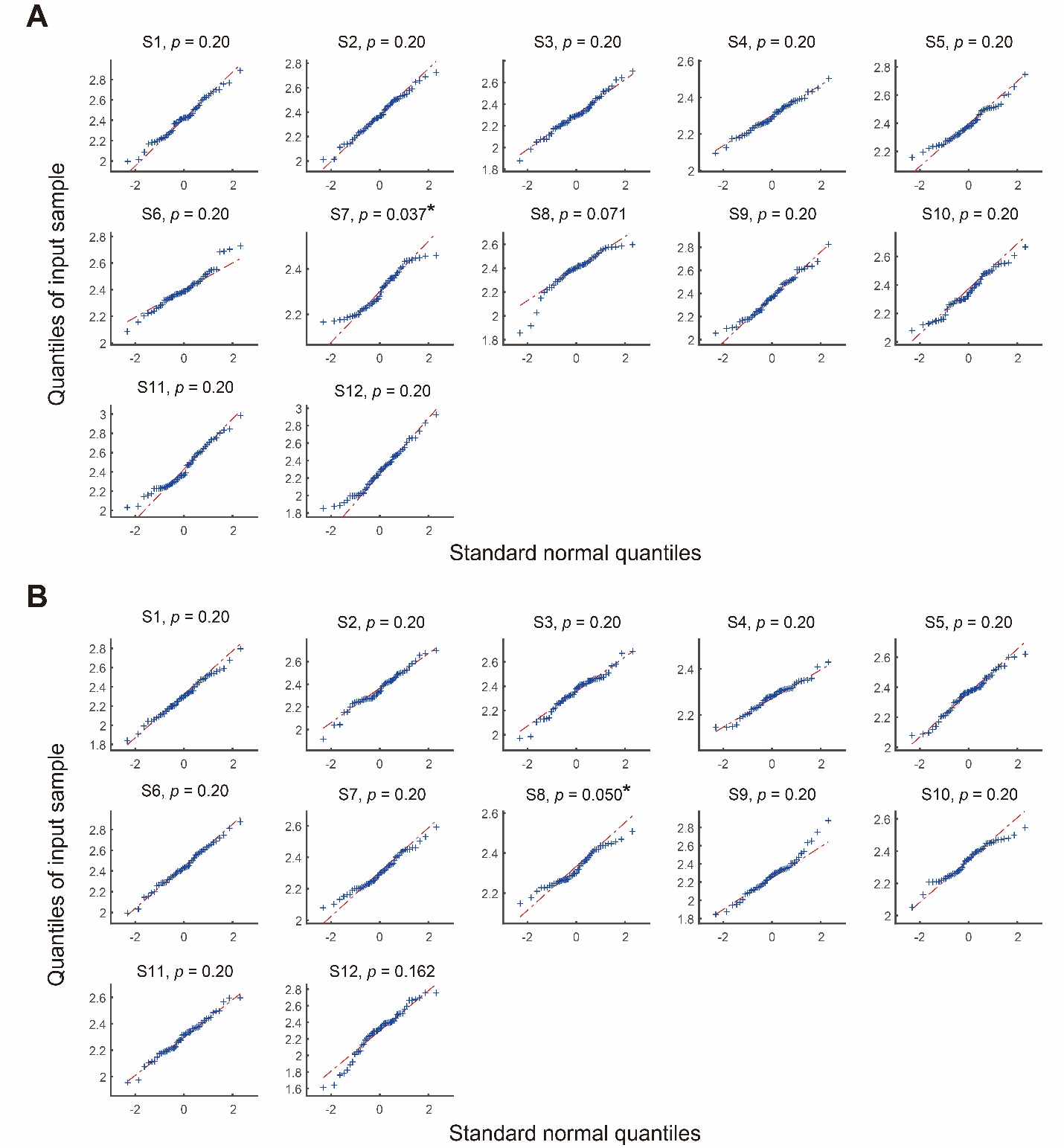
Supplementary Figure 3.** Q-Q plot for the response time and the result of Kolmogorov-Smirnov test in Experiment 2 (symmetric condition). Pre-test (A) and test during stimulation (B) in right anodal/left cathodal condition.

# Risk-Attitude Analysis in Experiment 2

We further conducted the risk-attitude (difference between the observed mean response time and the optimal mean response time) analysis in Experiment 2. To do so, we first calculated the optimal response time in Experiment 2. Because the gain function in Experiment 2 was the symmetric (Figure 1D), responding at 2300 ms (i.e., maximum gain) was the optimal strategy for all the participants and all the stimulation conditions. We then calculated the risk-attitude as $T_{obs}- T_{opt}$. We performed two-way within-subject ANOVA using two experimental blocks (pre-test and test during stimulation) and three stimulation protocols (R anodal/L cathodal, R cathodal/L anodal, and sham) as independent variables. Neither main effect of stimulation protocol (*F* [2, 22] = 0.44, *p* = 0.65, $\eta^{2}$ = 0.02) nor significant interaction (*F* [2, 22] = 1.32, *p* = 0.29, $\eta^{2}$ = 0.04) were found. In the R anodal/L cathodal condition, there was no significant difference between the test during stimulation (26 ± 14 ms, mean ± sem) and the pre-test (59 ± 15 ms) (*p* = 0.078, Supplementary Fig. 4) and between the training (14 ± 13 ms) and the test during stimulation (two-tailed paired *t* test: *t* [11] = −0.64, *p* = 0.54, Supplementary Figure 4).


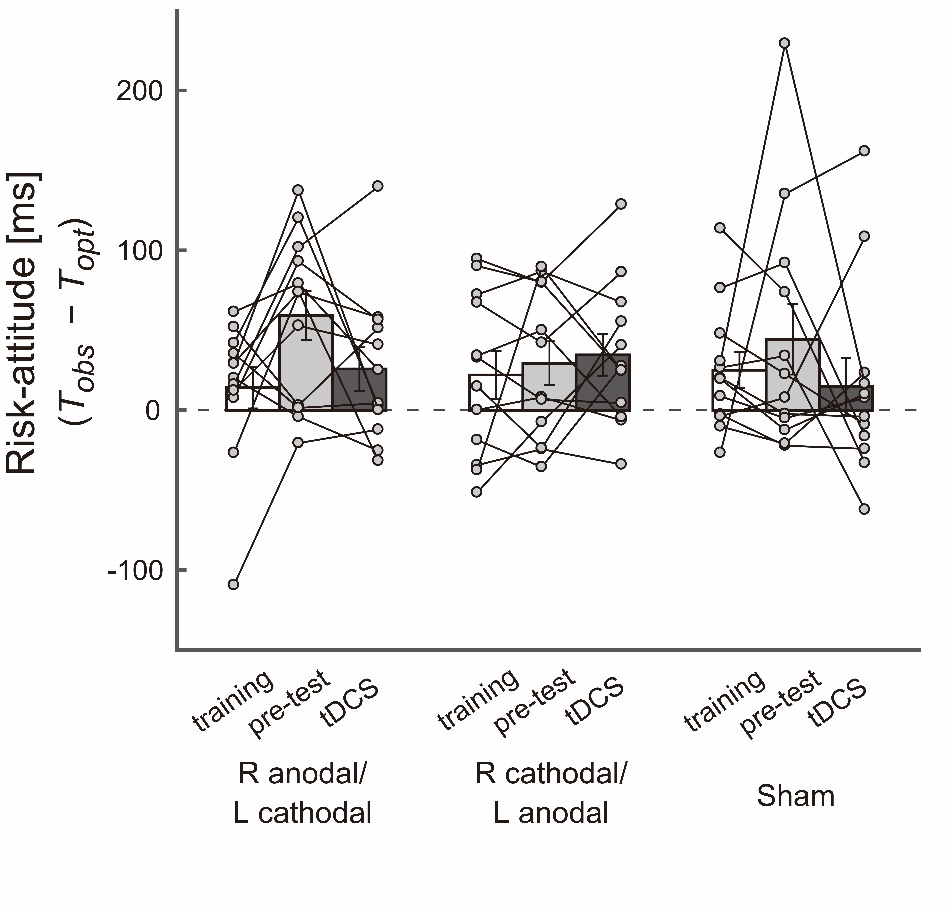


**Supplementary Figure 4.** Behavioral performance in the symmetric condition. The average risk-attitude across the participants is plotted. The *X*-axis indicates experimental blocks (training, pre-test, and test during stimulation) in each stimulation protocol.
